# Bloodstream infections among patients receiving therapeutic plasma exchanges in the intensive care unit: a 10 year multicentric study

**DOI:** 10.1186/s13613-024-01346-7

**Published:** 2024-07-29

**Authors:** Sofiane Fodil, Tomas Urbina, Swann Bredin, Julien Mayaux, Antoine Lafarge, Louaï Missri, Eric Maury, Alexandre Demoule, Frederic Pene, Eric Mariotte, Hafid Ait-Oufella

**Affiliations:** 1 Service de Médecine Intensive-Réanimation, Réanimation Médicale, Hôpital Saint-Antoine, Assistance Publique-Hôpitaux de Paris, Sorbonne Université, 184 rue du Faubourg Saint-Antoine, 75571 Paris Cedex 12, France; 2 Service de Médecine Intensive-Réanimation, Hôpital Cochin, Assistance Publique-Hôpitaux de Paris, Paris, France; 3 Service de Médecine Intensive-Réanimation, Groupe Hospitalier Pitié-Salpétrière, Assistance Publique-Hôpitaux de Paris, Paris, France; 4 Service de Médecine Intensive-Réanimation, Hôpital Saint-Louis, Assistance Publique-Hôpitaux de Paris, Paris, France; 5 Inserm U970, Cardiovascular Research Center, Université Paris-Cité, Paris, France

**Keywords:** Therapeutic plasma exchange, Intensive care unit, Critical care, Bloodstream infections, Intra-vascular catheter infection, Health-care acquired infections

## Abstract

**Background:**

Therapeutic plasma exchanges (TPE), which affect the humoral response, are often performed in combination with immunosuppressive drugs. For this reason, TPE may be associated with an increased susceptibility to infections. We aimed to describe blood stream infection (BSI) incidence in ICU patients treated with TPE and to identify associated risk factors.

**Methods:**

We retrospectively included patients that had received at least one session of TPE in the ICU of one of the 4 participating centers (all in Paris, France) between January 1st 2010 and December 31th 2019. Patients presenting with a BSI during ICU stay were compared to patients without such an infection. Risk factors for BSI were identified by a multivariate logistic regression model.

**Results:**

Over 10 years in the 4 ICUs, 387 patients were included, with a median of 5 [2–7] TPE sessions per patient. Most frequent indications for TPE were thrombotic microangiopathy (47%), central nervous system inflammatory disorders (11%), hyperviscosity syndrome (11%) and ANCA associated vasculitis (8.5%). Thirty-one patients (8%) presented with a BSI during their ICU stay, a median of 7 [3–11] days after start of TPE. In a multivariate logistic regression model, diabetes (OR 3.32 [1.21–8.32]) and total number of TPE sessions (OR 1.14 [1.08–1.20]) were independent risk factors for BSI. There was no difference between TPE catheter infection related BSI (n = 11 (35%)) and other sources of BSI (n = 20 (65%)) regarding catheter insertion site (p = 0.458) or rate of TPE catheter related deep vein thrombosis (p = 0.601). ICU course was severe in patients presenting with BSI when compared to patients without BSI, with higher need for mechanical ventilation (45% *vs* 18%, p = 0.001), renal replacement therapy (42% *vs* 20%, p = 0.011), vasopressors (32% *vs* 12%, p = 0.004) and a higher mortality (19% *vs* 5%, p = 0.010).

**Conclusion:**

Blood stream infections are frequent in patients receiving TPE in the ICU, and are associated with a severe ICU course. Vigilant monitoring is crucial particularly for patients receiving a high number of TPE sessions.

**Supplementary Information:**

The online version contains supplementary material available at 10.1186/s13613-024-01346-7.

## Background

Therapeutic Plasma Exchanges (TPE) can be required in an array of diseases potentially associated with critical illness [1]. Moreover, even in the absence of a critical condition, patients may be admitted in ICU for TPE as it is an invasive procedure requiring an experienced staff with specific equipment and close monitoring [2]. TPE are often performed in combination with immunosuppressive drugs such as high doses corticosteroids or Rituximab. In addition, TPE might impair the humoral response by depletion of endogenous immunoglobulins [3, 4]. Consequently, TPE may be associated with an increased risk for infections.

More specifically, due to impaired immune responses and the need for a vascular access mainly by central venous catheters, the occurrence of a blood stream infection (BSI) is a matter of concern when performing TPE. BSI represent 20% of ICU-acquired sepsis and are associated with poor outcome, while early adequate antimicrobial therapy and source identification and control are cornerstones of management [5]. Epidemiology can vary widely according to case-mix [5], and patients receiving TPE have unique characteristics that could affect incidence, source, microbiology and outcome.

Data regarding the infectious risk associated with TPE are scarce and not concluding in non-critically ill patients. Early small case series of patients with ANCA-associated vasculitis showed 63% rates of severe infections in patients treated with TPE, compared to 9,5% in patients with similar disease treated without TPE, while more recent large-scale randomized trials found no differences between two such groups [6, 7]. Data is scarce in critically-ill patients, but a recent cohort suggested that TPE use in patients admitted to the ICU was associated with a higher risk of infectious complications [8].

The aim of our multicentric retrospective study was to describe BSI among patients receiving TPE in the ICU, and to identify risk factors for these infections in this specific population.

## Methods

### Patients

We retrospectively included patients that had received at least one session of TPE in the ICU of one of the 4 participating centers (all in Paris, France) between January 1st 2010 and December 31th 2019, a recent period during which we could access detailed patient medical records, and before the COVID-19 outbreak. Patients were identified by searching hospital databases for ICD-10 code FEJF0020 according to the International Classification of Diseases (10th version). Local investigators reviewed all medical files and included all patients receiving at least one TPE session during their ICU stay, with no further selection or exclusion criteria. These patients were counted once in the analysis, whatever the number of TPE sessions performed. Patients presenting with a bloodstream infection (BSI patients) (defined as a positive blood culture requiring treatment according to the treating physician) during ICU stay were compared to patients without such an infection (No-BSI patients). Patients with a positive blood-culture not consistent with BSI were considered as contaminations and excluded from analysis. Among bacteremic patients, those with TPE catheter infection as the source of infection were compared to those with another source of infection.

### Data collection

We collected epidemiological, clinical and laboratory characteristics, treatment and outcome data from electronic medical records. Data collected upon admission included age, gender, comorbidities, immunosuppressive drugs and disease severity as evaluated by the Simplified Acute Physiology Score II (SAPS II).

We recorded indication for PE, insertion site of dedicated intravascular catheter, total number of sessions performed and percentage of fresh frozen plasma used as fluid substitution.

Regarding BSI, we collected time from start of TPE to first positive blood culture (identical to time from catheter insertion as TPE sessions were started on day of catheter insertion), temperature on the day of bacteremia, suspected source, microbiology and duration of bacteremia. The presence of TPE catheter related deep vein thrombosis and results of TPE catheter culture after removal were likely recorded. TPE catheter related blood stream infection (CRBSI) was defined according to most recent guidelines [9].

Recorded outcomes included ICU mortality, ICU and hospital length of stay and need for organ support during ICU stay.

### Statistical analysis

Patient characteristics were summarized as median (25–75th percentiles) for continuous variables with skewed distributions and percentages for categorical variables as appropriate. Differences between groups were compared for continuous variables using the Mann–Whitney test, and Fisher’s exact test for categorical variables. To identify risk factors for BSI, variables with a p-value < 0.05 by univariate analysis were included in a multivariate logistic regression model. The final model was obtained after gradual removal of non-significant factors in the model (p value ≥ 0.05). All tests were two-sided, and *p* values less than 0.05 were considered statistically significant. No imputation was performed for missing data due to < 10% of missing data. Statistical analyses and graphical representations were performed using GraphPad Prism 5.04 (Graph Pad Software Inc. ®).x

### Study approval

This study was approved by the ethical committee of the French society of Intensive Care (reference CE SRLF 21–90). The study was performed in accordance with Good Clinical Practice and the Declaration of Helsinki principles for ethical research. All patients received information during their hospital stay that data abstracted from their medical charts could be used for research. Data was collected and anonymized according to the requirements of the *Commission Nationale de l’Informatique et des Libertés* (N° 2,232,515).

## Results

### Study population

**Fig. 1:**
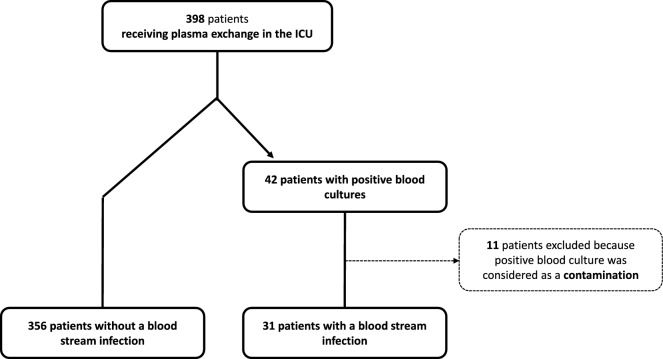
Study Flow chart. Contaminations were defined as any positive blood cultures not requiring treatment (antibiotic treatment or catheter removal) according to treating physician

Over 10 years in the 4 ICUs, 387 patients that had received a median of 5 [2–7] sessions of TPE were included (Fig. 1). Median age was 49 [34–63] years, with 60.5% of women. Most common comorbidities were hematological malignancies (15%) and diabetes (11%). Median SAPS II was 26 [13–39]. Most frequent indications for TPE were thrombotic microangiopathy (47%), central nervous system inflammatory disorders (11%), hyperviscosity syndrome (11%) and ANCA associated vasculitis (8.5%). Upon admission, 86% of patients received corticosteroids and 21% at least one adjunct immunosuppressive drug (of which the most common were rituximab (n = 57), cyclophosphamide (n = 21), polyvalent immunoglobulins (n = 17) and vincristine (n = 12)). At least one organ support therapy (renal replacement therapy, mechanical ventilation or vasopressor infusion) was required for 124 (32%) patients.

### Characteristics of BSI episodes and outcome

**Fig. 2:**
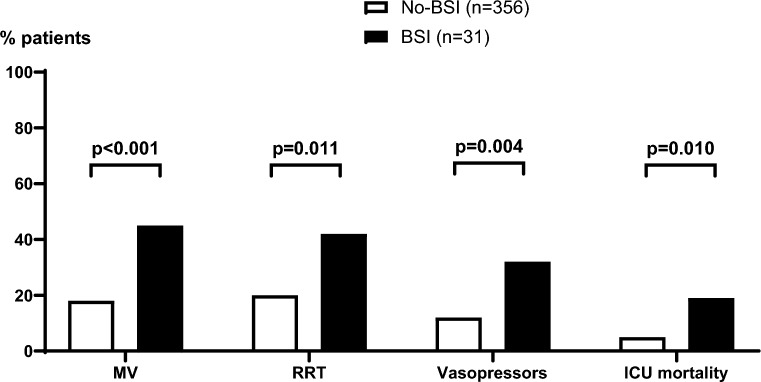
Outcomes of patients according to the occurrence of a blood-stream infection during ICU stay. BSI: blood stream infection. MV: mechanical ventilation. RRT: renal replacement therapy. ICU intensive care unit. p-value for Fisher’s exact test for categorical variables and Mann–Whitney’s test for continuous variables

Out of 42 positive blood cultures recorded, 11 were considered contaminations and excluded (Fig. 1). Finally, we identified 31 patients (8%) presenting with a BSI during their ICU stay, a median of 7 [3–11] days after start of TPE. All but 5 (16%) patients were febrile on day of first positive blood culture. ICU course was severe in patients presenting with BSI compared to others, with higher need for mechanical ventilation (45% *vs* 18%, p = 0.001), renal replacement therapy (42% *vs* 20%, p = 0.011), and vasopressors (32% *vs* 12%, p = 0.004) (Fig. 2). ICU mortality rate was higher in patients with BSI (19% *vs* 5%, p = 0.010), with ICU and hospital length of stay of respectively 17 [10–24] *vs* 6 [4–10] (p < 0.001) and 34 [20–48] *vs* 17 [12–26] (p < 0.001) days.

### Risk factors for BSI

There was no significant difference between patients experiencing BSI and others regarding corticosteroid treatment (84% *vs* 86%, p = 0.797), malignant hemopathy (13% *vs* 16%, p = 0.801), percentage of fresh frozen plasma used for TPE (100 [30–100]% *vs* 100 [0–100]%, p = 0.406), TPE indication (p = 0.081) or disease severity at admission assessed by SAPSII (30 [17–45] *vs* 25 [13–39], p = 0.114) (Table 1). By univariate analysis, diabetes, body mass index, adjunct immunosuppressive drugs to steroids and total number of TPE sessions were the variables associated with the occurrence of BSI, while catheter insertion site was not. In a multivariate logistic regression model, diabetes (OR 3.32 [1.21–8.32]) and total number of TPE sessions (OR 1.14 [1.08–1.20]) remained independent risk factors for BSI.

**Table 1:** Study population admission characteristics and outcome

	No-BSI patients (n = 356)	BSI^a^ patients (n = 31)	P-value
Demographics			
Age, years, median [IQR^*b*^]	49 [34–63]	48 [34–61]	0.975
Male sex, n (%)	146 (41%)	7 (33%)	0.054
Comorbidities, n (%)			
Body mass index > 30 kg/m^2^	25 [22–28]	26.5 [22.6–32.7]	0.049
Diabetes mellitus	34 (10%)	7 (23%)	0.034
Malignant hemopathy	56 (16%)	4 (13%)	0.801
Steroids	302 (86%)	26 (84%)	0.797
Other Immunosuppressive drugs	103 (29%)	18 (58%)	0.002
TPE^c^ indication, n (%)			0.081
Thrombotic microangiopathy	172 (48%)	14 (45%)	
ANCA^d^ associated vasculitis	33 (9%)	1 (3%)	
CNS^e^ inflammatory disease	40(11%)	2 (6%)	
Hyperviscosity	39 (11%)	1 (3%)	
Other	72 (20%)	13 (42%)	
Severity upon admission			
SAPS II^f^, median [IQR])	25 [13–39]	30 [17–45]	0.114
Total number of TPE sessions, median [IQR]	5 [2–7]	7 [5–14]	< 0.001

^a^BSI: bloodstream infection

^b^IQR: interquartile range

^c^TPE: therapeutic plasma exchange

^d^ANCA: Antineutrophil cytoplasmic antibodies

^e^CNS: central nervous system

^f^SAPS II: simplified acute physiology score II. Other indications for TPE (n = 85) included catastrophic antiphospholipd syndrome (n = 16), lupus (n = 15), autoimmune cytopenia (8), cryoglobulinemia (7), myositis (n = 6), acute polyradiculonevritis (n = 6), acute graft rejection (n = 4), HLA desentization (n = 4), polyarteritis nodosa (n = 3), hypertriglyceridemia (n = 3), myxoedema (n = 2), acute glomerulonephritis (n = 2), intoxication (n = 1), myasthenia gravis (n = 1), botulism (n = 1), acute renal scleroderma crisis (n = 1), Susac syndrome (n = 1), Henoch-Schonlein disease (n = 1), capillary leak syndrome (n = 1), schizophrenia (n = 1), iatrogenic thyrotoxicosis (n = 1), pulmonary-renal syndrome (n = 1)

### Comparison of TPE catheter infection to BSI from other source

Of 31 BSI episodes, 11 (35%) were identified as TPE catheter related, 20 (65%) as from another source of infection (7 of unknown origin, 5 other intra-vascular device infection, 4 respiratory tract infections, 1 intra-abdominal infection, 1 surgical site infection, 1 urinary tract infection, 1 skin and soft tissue infection) (Table 2). On univariate analysis, there was no statistically significant difference between TPE catheter infection related BSI and other sources of infection regarding catheter insertion site (p = 0.458) or rate of TPE catheter related deep vein thrombosis (p = 0.601). No baseline parameter was significantly different between the two groups, though there was a non-significant trend towards longer time from start of TPE to BSI in patients with TPE catheter infection (9 [4–13] *vs* 5 [1–10] days, p = 0.101) (Supplemental Fig. 1). Infections were mainly monomicrobial (90%), with a predominance of gram-negative bacilli (45% Enterobacteriaceae, 7% non-fermenting gram negative bacilli). There was a higher rate of MSSA infections in TPE catheter related infections (36% *vs* 5%, p = 0.042), while MRSA or fungal infections were only seen in patients with other sources of infection.

**Table 2:** Characteristics of bloodstream infections according to source of infection

	BSI^*a*^ patients (n = 31)	TPE Catheter related (n = 11)	Other source (n = 20)	P-value
Time from start of TPE^b^ and bacteriemia, days, median [IQR]^c^	7 [3–11]	9 [4–13]	5 [1–10]	0.101
Temperature on day of bacteriemia, °C, median [IQR]	38.5 [38–39]	38.5 [37.3–39]	38.5 [38–39]	0.596
Patients with temperature < 38 °C on day of bacteriemia, n (%)	5 (16%)	2 (18%)	3 (15%)	> 0.99
TPE Catheter insertion site, n (%)				0.458
Jugular	16 (52%)	7 (64%)	9 (45%)	
Femoral	15 (48%)	4 (36%)	11 (55%)	
TPE Catheter related deep vein thrombosis, n (%)	4 (13%)	2 (18%)	2 (10%)	0.680
Duration of bacteriemia, days, median [IQR]	2 [1–2.5]	2 [1–2]	2 [1- 3]	0.678
Positive TPE catheter culture	9 (29%)	8 (73%)	1 (5%)	< 0.001
Microbiology, n (%)				
MSSA^d^	5 (16%)	4 (36%)	1 (5%)	0.042
Enterobacteriacae	14 (45%)	5 (46%)	8 (40%)	> 0.99
MRSA^e^	3 (10%)	0	3 (15%)	0.535
Fungi	3 (10%)	0	3 (15%)	0.535
Non-fermenting GNB^f^	2 (7%)	1 (9%)	1 (5%)	> 0.99
Others	4 (14%)	1 (9%)	3 (15%)	> 0.99
Polymicrobial	2 (7%)	1 (9%)	2 (10%)	> 0.99

^a^BSI: bloodstream infection

^b^TPE: plasma exchange

^c^IQR: interquartile range

^d^MSSA: methicillin-susceptible Staphylococcus aureus

^e^MRSA: Methicillin-Resistant Staphylococcus aureus

^f^GNB: gram-negative bacilli

## Discussion

In this large multicenter retrospective cohort of patients receiving TPE in the ICU, BSI occurred in 8% of patients and was associated with a more severe disease course than patients without BSI. Risk factors for BSI on multivariate analysis were diabetes and total number of TPE sessions. One third of BSI episodes originated from a TPE catheter infection, regardless of catheter insertion site or occurrence of catheter-related deep vein thrombosis.

The 8% incidence of BSI is on the higher end of the reported ICU acquired BSI incidence of 5–7% [5, 10], and median time to BSI was slightly shorter than in the EUROBACT-2 study (7 [3–11] days *vs* 13 [8–25] days) [11]. This was anticipated in a population with two known risk factors for ICU acquired BSI de facto (*i.e.* immunosuppression and invasive devices) [5], highlighting the need for a high index of suspicion for BSI in patients receiving TPE in the ICU. Nevertheless, 84% of patients experiencing BSI were febrile on the day of infection, despite the use of immunosuppressive drugs such as steroids which may attenuate pyrogenic response [12].

Little is known about infectious complications in critically-ill patients treated by TPE [8], but a recent work including 124 patients suggested a higher rate of overall infectious complications when compared to critically-ill patients not receiving TPE. The rate of BSI was 15%, with, similarly to our results, 33% originating from a TPE catheter infection. Risk factors for infectious complications they identified were length of ICU stay and need for mechanical ventilation, two known generic risk factors for infection in critically-ill patients [13]. In this larger study focusing only on BSI rather than all infections, the number of TPE sessions was identified as a more specific independent risk factor. Interestingly, while depletion in humoral components of the immune system has been suggested as a mechanism for the increased risk of infection associated with TPE [3, 4], the percentage of fresh frozen plasma (containing both immunoglobulins and complement) used as a substitute for TPE in our work was not associated with occurrence of BSI. A bias due to TTP being the most frequent indication for TPE in our cohort, with a large number of patients receiving 100% of fresh frozen plasma as a substitution fluid or a lack of statistical power cannot be excluded. Alternatively, the mechanism by which TPE could increase susceptibility to infection may be more complex than just humoral immune components depletion. Although the number of TPE sessions is likely associated with underlying disease’s severity, neither treatment by corticosteroids alone (though we could not assess the impact of corticosteroid dose) or with an adjunct immunosuppressive drug, both reflections of disease severity, were associated with the occurrence of a BSI. Finally, number of TPE sessions was strongly correlated to time from catheter insertion, a known risk factor for BSI.

Though sources of BSI were similar to those found in the EUROBACT-2 study [11], with as expected a slightly higher rate of catheter infections (35% *vs* 26.4%), the rate of BSI associated with TPE catheter infection in our cohort was higher than previously reported in non-critically ill patients receiving TPE [14], highlighting the specificities of this population. A probable explanation is the prevalent use of peripheral venous access (72%) for TPE in this previous cohort of non-critically-ill patients, though use of fresh frozen plasma as a substitutive fluid was also scarcer and details on associated immunosuppressive therapy were not described [14].There was no difference between TPE catheter infection and BSI from other sources, particularly in catheter insertion site or catheter related deep vein thrombosis, consistent with previous work [8]. Thus, when facing an episode of BSI in a patient treated with TPE in the ICU, clinical work-up should remain broad to identify the source of infection.

This work has some limitations, mainly the inherent bias associated with its retrospective design, with potential biases in patient selection and outcome assessment. The management or diagnosis work-up for BSI and BSI source was not standardized, nor was management of underlying disease (*i.e.* choice and dosing of immunosuppressive drugs), and we cannot excluded unmeasured or unknown confounding variables despite our multivariate analysis.. Results regarding outcome should thus be considered exploratory and interpreted with caution. Though we cannot exclude a lack of statistical power to identify certain risk factors for BSI such as degree of immunosuppression or indication for TPE, this is to our knowledge the largest multicenter study reporting on infectious complications among patients receiving TPE in the ICU. Although generalizability could be affected by the unique case mix of our four ICUs in the Paris area, our study reflects the real-life practice of TPE in the ICU for a wide array of conditions, providing important descriptive data on BSI occurrence in these patients. Our results advocate for a vigilant monitoring for BSI in patients receiving TPE in the ICU, particularly in diabetic patients with a high number of TPE sessions. Further prospective studies including other patient-centered outcomes of the therapy such as underlying disease control are needed in order to inform clinical management.

## Conclusions

In this multicenter retrospective cohort of patients receiving TPE in the ICU, BSI was frequent and associated with a severe ICU course. Vigilant monitoring is crucial particularly for diabetic patients or those receiving a high number of TPE sessions. This findings should be assessed prospectively along with other patient centered outcomes of the therapy to inform clinical management.

### Supplementary Information

Supplementary file1.

## Data Availability

The dataset used for the analysis is available from the corresponding author on reasonable request.
